# The Influence of Neocate in Paediatric Short Bowel Syndrome on PN Weaning

**DOI:** 10.1155/2010/297575

**Published:** 2010-05-31

**Authors:** E. De Greef, T. Mahler, A. Janssen, H. Cuypers, G. Veereman-Wauters

**Affiliations:** Queen Paola Children's Hospital ZNA, Antwerp, Belgium

## Abstract

Clinical management of short bowel syndrome remains a multistage process. Although PN is crucial, early introduction of enteral feeding is mandatory. We describe retrospectively 4 patients with an ultrashort bowel who could be weaned off PN on very short terms after introduction of an amino-acid-based formula (Neocate). Patient 1 had congenital short bowel with 50 cm small bowel and 30 cm colon. He had persistent diarrhoea on a semielementary formula. When Neocate was introduced he could be weaned from PN within 6 months. Patient 2 needed multiple surgical interventions because of NEC at D 27. He maintained 40 cm small bowel and an intact colon and remained PN dependent on semielemental formula. After introducing Neocate, PN could be weaned within 3 months. In the next 2 patients, Neocate was introduced as initial enteral feeding after bowel resection following antenatal midgut volvulus. Patient 3 had 20 cm small bowel and an intact colon. PN was weaned after 2 months. Patient 4 had 9 cm small bowel and an intact colon. PN was weaned after 13 months. In all patients Ileocaecal valve (ICV) was preserved. No consensus is reached on the type of formula to use for short bowel syndrome. Compared to recent data in the literature, the weaning period in these 4 patients was significantly shortened on an aminoacid based formula. The reason for this may lie in the antiallergic properties of this formula. We recommend the use of an amino-acid-based formula to induce earlier weaning of PN.

## 1. Introduction

Short Bowel Syndrome (SBS) is the most common cause of protracted intestinal failure. About 85%–94% of SBS infants survive the neonatal period [1]. Even though the causes of SBS are diverse, the management has identical pillars: parenteral nutrition (PN) and early enteral nutrition (EN). 

Prognosis is associated with age-adjusted small bowel length, the presence of the ileocaecal valve (ICV) and colon, the advent of PN complications, and the capacity of the remaining intestine to adapt [2]. Several hormonal and surgical methods to improve bowel adaptation have been reported [3–8], but EN remains an important and controllable factor. Direct trophic effect of nutrients and stimulation of gastrointestinal secretions and hormonal factors enhances intestinal adaptation [3, 6, 8]. We present 4 cases of ultrashort bowel syndrome (<40 cm residual small bowel) (Table 1) rapidly weaned of PN after introduction of an elemental formula (Neocate, *Nutricia, Strombeek-Bever, Belgium*). Neocate is the only elemental formula available in Belgium. Table 2 illustrates the composition of Neocate compared to other elemental formulas available in other countries and to the semi-elementary formula used in 2/4 patients. All patients received a PPI (Omeprazole) as additional medication as part of the SBS treatment [9].

## 2. Case Reports

### 2.1. Patient 1

The diagnosis of congenital short bowel in Patient 1 was made by explorative laparotomy at the age of 3 months. He had no Treitz angle, a small bowel of 50 cm, and a colon of 30 cm length. Postoperatively, he received PN. EN with semi-elemental formula was started following the EN scheme used in our department as illustrated in Table 3. He was discharged from the hospital at 8 months. A third of his calories was tolerated by continuous EN through a gastrostomy (43 kcal/kg/d), and home PN was organised for the remaining caloric requirements (86 kcal/kg/d). The advancement of EN was limited by recurrent profuse diarrhoea, and at 3 years of age, Neocate was introduced. He was started at 1/3 of his daily requirements (total cals: 130 kcal/kg/d) on continuous enteral infusion and upgraded following Table 2. Step 2 was reached 8 months later and PN could be discontinued. After initial PN weaning, the patient needed another 5 days of TPN during an episode of D-lactate acidosis with severe dehydration. He was started on probiotics (*VSL3: 450 bilion lactic acid bacteria per package in defined ratios of bifidobacterium breve, bifidobactium longum, bifidobacterium infantis, lactobacillus acidophilus, lactobacillus plantarum, lactobacillus paracasei, lactobacillus bulgarius, streptococcus thermophilus by VSL Pharmaceuticals, Inc.*) and remains well since. Introduction of soy and cow's milk protein was tried at 6 years. Although RAST testing remained negative, serum albumin dropped, and the patient stopped growing for 12 months (25th percentile to 10th percentile). Biochemistry and growth, following the 10th percentile, recovered on reintroduction of Neocate. Presently, the boy is 9 years old. He remains on step 4 of the weaning scheme (Table 2) receiving 46% of his calories by nocturnal feeds (1500 mL neocate 20% + 20 g protifar = 1527 kcal/58.5 g EW/69.2 g V/168 g KH), and reintroduction of cow's milk and soy protein is planned. His motor development is normal, and his growth parameters (height and weight) follow the 10th percentile.

### 2.2. Patient 2

Patient 2 was born at 25 weeks gestation, with a birth weight of 700 g and suffered severe enterocolitis at day 27. It led to resection of a large part of the small bowel. A second look operation revealed 32 cm of small bowel remaining to re-establish continuity. The ICV and colon were preserved. Several attempts to start and increase EN with semi-elemental formula (*Alfare, Nestle, Brussels, Belgium*) failed. The patient was transferred to our unit at the age of 4 months. Continuous enteral feeding with Neocate was started successfully in combination with PN and oral stimulation (Table 2, step 1). At the age of 5 months, she developed severe diarrhoea and abdominal distension. A proximal jejunal stenosis was repaired with minimal bowel resection. Five days after surgery, EN was restarted and successfully upgraded following Table 1. PN was stopped 3 months after the initiation of Neocate. The patient reached step 2 (Table 2) when she left the hospital at 8 months of age (total calories 128.4 cal/kg/day). The further follow-up was performed in the referring centre. She obtained complete oral gastrointestinal autonomy at the age of 1 year and has successfully been switched to semi-elemental formula (*Nutramigen, Mead Johnson, Braine-L'alleud, Belgium and Frisomel; Friesland Coberco Dairy foods, Meppel, the Netherlands*). She is treated with a proton pomp inhibitor because of severe gastroesophageal reflux. Her growth is normal (weight 10th percentile, height 10th–25th percentile), and her vitamin status and electrolytes are checked regularly and are within the normal range without supplements.

### 2.3. Patient 3

Patient 3, born at 33 weeks, 1700 g, had dark gastric aspirates five days after birth. An urgent laparotomy showed a midgut volvulus. A large bowel resection left 12 cm of jejunum and 8 cm of ileum with an intact colon and a preserved ICV. Postoperatively the patient received total PN. EN with Neocate was started 14 days after surgery following Table 2. At the age of 2 months, the patient tolerated full EN on Neocate 20% (step 2, Table 2). A gastrostomy was placed, and PN was discontinued. At discharge, he was on daytime boluses and night-time continuous feeding (step 3a Table 2) which were adapted weekly following tolerance (total calories 142 cal/kg/day, 10% po, 90% ps). He achieved oral autonomy (step 4 Table 2) at 6 months, and solids were introduced. His oral caloric intake from Neocate remained 93 cal/kg/day. By that time, the patient caught up in length and weight, reaching normal growth percentiles (3rd percentile) for his age.

### 2.4. Patient 4

In patient 4, a duplicature of the stomach was suspected on prenatal ultrasound. Born at term, the amniotic fluid was meconium stained, the abdomen was dilated, and vital signs were compromised. An explorative laparotomy showed necrotising small bowel with hemorrhagic fluid, suggesting prenatal volvulus. Extensive resection was needed, leaving 5 cm of duodenum, 4 cm of terminal ileum, the ICV, and the colon. Postoperatively TPN was initiated. On day 12, continuous enteral feeding with Neocate following Table 2 was started. A gastrostomy was placed at the age of 1 month. When she was discharged home at the age of 3 months, the gastrostomy had been removed due to peritonitis, and she was fed by small oral boluses of Neocate 20% (6 × 20 mL) and nightly PN (130 kcal/kg/d, 36% po and 64% PN). Although she made good progress with oral Neocate, growth deviation from the 50th to the 25th percentile indicated the need for extra support and nocturnal feeding through a gastrostomy at 11 months. Two months later PN was discontinued. The patient maintained her growth with oral intake and supplemental enteral feeding (Total 121.5 kcal/kg/d, 73.5% po and 26.5% ps) (step 4 Table 2). 

Frequent liquid stools without reducing substances were debilitating and persisted despite trials of Cotrimoxazole, cholestyramin, and probiotics. Stools normalised spontaneously over time. The patient is now 3.5 years old. She eats a varied diet during the day and has nightly enteral supplements with Neocate (Total 121.5 kcal/kg/d, 48% po and 52% ps, 4.9 g prot/kg/d) (step 4, Table 2). Cow's milk and soy are excluded from her diet because of clinical intolerance and elevated RAST tests. 

She was toilet trained at 30 months and has normal motor development and growth (weight 50th percentile, height 25th percentile).

## 3. Discussion

SBS is defined as malabsorption and failure to thrive following extensive resection of the small bowel. Surgical classification of SBS is based on the residual length of the small intestine measured peroperatively along the antimesenteric border. Although residual length is an important element, it is not the only prognostic factor. The adaptive capacity of the remaining gut is a major component for obtaining enteral autonomy. Enteral autonomy and PN weaning while maintaining adequate growth are the principal endpoints in this group of patients. Early introduction of EN is universally recommended. The scheme for EN used in our centre is illustrated in Table 3. Continuous enteral feeding is the starting method of choice as it enhances intestinal absorption and improves tolerance [10]. Concentration of the continuous feed is progressively increased until total caloric requirements are tolerated. Only then, PN is discontinued, and bolus feeding initiated. Enteral tolerance is monitored by intake, growth, stool frequency, stool consistency, reducing substances, and biochemistry. Three of the 4 infants reported have an ultrashort gut (<40 cm), and one has a congenital short bowel of 50 cm, in which function is known to be altered [11]. All patients needed parenteral nutrition, but enteral autonomy was obtained and maintained relatively rapidly (<13 months) after introduction of Neocate (Table 1). 

Georgeson and Breaux [12] retrospectively described a group of 52 SBS patients receiving an elemental diet. Residual bowel length in 21/52 was <40 cm. PN weaning was obtained in 12/21, and the mean weaning period was 24.1 months. Not all patients could be weaned off PN, but the majority of this group had no ICV (17/21). Recurrence of PN after initial weaning was not recorded. More recently, Goulet et al. [1] reported 78 SBS patients, receiving initially a hydrolyzed diet, followed up for 15 years. Ultrashort bowel, <40 cm, was seen in 21/78 patients. In 9 patients (42.8%) PN weaning failed, and in the 12 patients remaining (57.2%), enteral or parenteral support was necessary after initial PN weaning. Initial PN duration in those 12 patients was 47.4 ± 23.8 months. The weaning period in Georgeson's study was considerably shorter with an elemental diet compared with Goulets' group, receiving a semi-elemental diet (24.1 versus 47.4 months). 

There is no agreement in literature on what formula to use in SBS infants. Semi-elemental or elemental formulas are most frequently started [1, 6]. Where some studies suggest that a more complex diet with long chain fat (LCF) and complex carbohydrates enhances adaptation [13–15], others support the important influence of an elemental diet on the weaning of PN and enteral tolerance [16]. The first 2 patients described in our series failed to thrive on a hydrolyzed formula as did 4 patients reported by Bines et al. [17]. Enteral autonomy was obtained after introduction of elemental formula in all 6 patients. Although the ratio is different, both hydrolyzed and elemental formulas contain LCF and complex carbohydrates. Therefore, another contributing factor needs to be considered: the elimination of a certain allergen or the presence of a yet unidentified stimulating substance [17].

Many children acquire SBS at a very young age; it makes them naturally prone for cow's milk intolerance (CMI). In infants, cow's milk protein is the most frequent allergen. Generally, CMI is considered to be 2%-3% below the age of 2 years [18], but there is no exact data about the incidence of CMI and food allergy in SBS patients, except for small case series [16, 17]. 

An alteration in gut permeability, often present in SBS patients, can be a predisposing factor. First of all, the administration of TPN without enteral support increases intestinal permeability, whereas the administration of even small amounts of EN can reverse this process [19]. Bacterial overgrowth on the other hand, a common complication of SBS, influences gut permeability through local gut inflammation [20]. 

We think that the combination of these factors might make SBS patients more prone for CMI. In 2 of our patients (patient 1 and 4), even the late introduction of cow's milk protein failed. This is an additional argument for the contribution of CMI to their initial feeding intolerance and explains the success of Neocate in these patients. 

Only 1 study compared intestinal permeability in SBS patients in a crossover study with hydrolized and whole protein formula [15]. Ten patients were included when 30% of enteral nutrition was tolerated, and no difference in intestinal permeability was found. Although measurement of intestinal permeability in this patient group while investigating tolerance to different formulas is of interest, no comment was made about PN weaning, 30% of enteral nutrition had to be tolerated before inclusion, the study period was rather short (60 days), and no washout period in between formulas was considered. 

Because of a parental choice, none of the patients received breast milk. CMI develops less frequently in breastfed babies, and breast milk has the additional trophic effect of probiotics and growth factors on the intestinal mucosa, beneficial to SBS patients [8, 18]. Unfortunately, continuous feeding and increasing calories with breast milk can be more challenging. 

We realise that, even after reporting these 4 cases, the published data about the relationship between residual bowel length, bowel adaptation, formula, CMI, and PN weaning remain scarce. In all our patients ICV and colon were preserved, which increases the chance of definite PN weaning [1, 2, 6]. 

Evidence from randomised controlled trials (RCTs) on this subject is lacking. Because of limited patient numbers, large RCTs are difficult to perform in a single centre and a multicentre approach is necessary. Measurement of intestinal permeability, growth, allergic parameters, and PN duration should be included. 

If breast milk is not an option, we would recommend an amino-acid based formula as the initial enteral feeding for SBS infants. It may lead to reduction in PN duration, cost, and complications and benefit the patients' quality of life. Despite all, it remains important as a doctor to focus on the outcome for the patient and to continue investigating if the initial treatment fails.

## Figures and Tables

**Table 1:** Shows for every patient the residual small bowel length (RSBL = residual length of the small intestine measured peroperatively along the antimesenteric border), the age at the start of Neocate (N), the age of complete PN, weaning and the total time on PN. In all patients the ileocaecal valve and colon were present. m = months; d = days.

	Start N	Stop PN	Total PN	RSBL
Patient 1	36 m	44 m	8 m	50 cm
Patient 2	4 m	7 m	3 m	32 cm
Patient 3	19 d	2 m	1 m 11 d	20 cm
Patient 4	12 d	13 m	12 m 18 d	10 cm

**Table 2:** Composition of the 3 commercialised elemental infant formulas and the hydrolized formula used in 2/4 patients. *Nutricia, Strombeek-Bever, Belgium, **Sandoz Nutrition Corporation, Minneapolis, US; ^†^Abbot Nutrition, Illinois, US; ^‡^Nestle, Brussels, Belgium.

	Neocate*	Vivonex Pediatric**	Elecare^†^	Alfare^‡^
Kcal	**100**	100	100	100
Protein (g)	**3.1**	3	3.1	3
Fat (g)	**4.5**	2.9	4.8	5.08
%MCT	**5%**	68%	33%	40%
Carbohydrates (g)	**11.7**	15.5	10.7	10.92

*VITAMINS*				
Vit A (IU)	**409**	312.5	273	350
Vit D (IU)	**59.9**	62.5	42	60
Vit E (IU)	**1.14**	3.7	2.1	2.5
Vit K (mcg)	**8.7**	5	6	8
Thiamin (mcg)	**92.6**	190	210	70
Riboflavin (mcg)	**137.8**	225	105	150
Vit B6 (mcg)	**123.5**	250	84.2	80
Vit B12 (mcg)	**0.26**	0.37	0.4	0.3
Niacin (mcg)	**1544**	2500	1680	1000
Folic Acid (mcg)	**10.2**	25	29.5	9
Pantothenic Acid (mcg)	**620**	625	421	450
biotin (mcg)	**3.1**	12.5	4.2	2
Vit C (mg)	**9.2**	12.5	9	10
Choline (mg)	**13.1**	25	9.5	10
Inositol (mg)	**23.3**	7.5	5.1	5

*MINERALS*				
Calcium (mg)	**124**	121.5	116	77
Phosphorus (mg)	**93.1**	100	84.2	52
Magnesium (mg)	**12.4**	25	8.4	12
Iron (mg)	**1.85**	1.25	1.5	1
Zinc (mg)	**1.66**	1.5	0.8	1
Manganese (mcg)	**90**	250	84	7
Copper (mcg)	**124**	150	105	80
Iodine (mg)	**15.4**	15	8.4	15
Selenium (mcg)	**3.7**	3.7	2.3	3.5
Chromium (mcg)	**3.5**	5.6	2.3	
Molybdene (mcg)	**4.7**	9.4	2.5	
Sodium (mg)	**37.3**	50	45	50
Potassium (mg)	**155.1**	150	150	125
Chloride (mg)	**77.2**	125	60	90

**Table 3:** EN scheme used and validated in our department.

Step 1.a					
			Day		Night
Oral stimulation (ORS)	+	+	+	+	
Enteral Nutrition: continuous (Neocate)	—*	—*	—*	—*	—*
Parenteral Nutrition: Continuous	—	—	—	—	—

Step 1.b					
			Day		Night
Oral stimulation (ORS)	+	+	+	+	
Enteral Nutrition: continuous	—*	—*	—*	—*	—*
Parenteral Nutrition					—

Step 2					
			Day		Night
Oral stimulation (ORS)	+	+	+	+	
Enteral Nutrition: continuous Neocate 20%	—	—	—	—	—

Step 3.a					
			Day		Night
Oral Nutrition (Neocate)	—	—	—	—	
Enteral Nutrition: daily boluses	—	—	—	—	—
Nocturnal continuous					

Step 3.b					
			Day		Night
Oral Nutrition (diverse)	—	—	—	—	
Enteral Nutrition: daily boluses (Neocate)	—	—	—	—	—
Nocturnal continuous					

Step 4					
			Day		Night
Oral Nutrition (diverse)	—	—	—	—	
Enteral Nutrition: daily boluses (Neocate)	—	—	—	—	
OR					
			Day		Night
Oral Nutrition (diverse)	—	—	—	—	
Enteral Nutrition: Nocturnal continuous					—

Step 5					
			Day		Night
Oral Nutrition	—	—	—	—	

Step 6					
Diversification oral nutrition					

*Progressive increase in concentration, 13%–15%–17%–20% until tolerance for total caloric requirements and growth are obtained.
